# Meeting of the Illinois State Medical Society

**Published:** 1852-06

**Authors:** N. S. Davis

**Affiliations:** Jacksonville


*Meeting of the Illinois State Medical Society.*

Messrs. Editors:—The third annual meeting of our State Medical Society has just closed, and I presume a very brief account of its doings would not be uninteresting to your readers, although the official proceedings will doubtless soon be published.

The members of the society assembled in the Old School Presbyterian Church, Jacksonville, at 11 o'clock A.M., on the 1st inst., and were called to order by the president, Dr. Samuel Thompson. A very respectable number of delegates and permanent members were present and answered to their names. After the usual preliminary business, the society adjourned until 2 P. M.

The afternoon session was devoted to the election of officers, the election of new members, and the transaction of miscellaneous business. Dr. R. Rouse, of Peoria, was elected President; Drs. T. Hall of Toulon, and E. English of Jacksonville, Vice-Presidents; Dr. E. Dickinson of Peoria, Treasurer; and Drs. E. S. Cooper of Peoria, and H. A. Johnson of Chicago, Secretaries. The usual standing committees were also appointed, and the city of Chicago selected as the place for holding the next annual meeting. The evening was spent in listening to a very interesting valedictory address, from the retiring president, Dr. Thompson. A large part of the second day was devoted to the reading of reports from standing committees and private papers connected therewith. The Chairman of the Committee on Practical Medicine and the Progress of Epidemics, made a report of considerable length, and embracing a paper on dysentery and pneumonia, read by Dr. T. Hall of Toulon, who was also a member of the committee. There being no regular report from the Committee on Surgery, Dr. E. S. Cooper of Peoria, read three short papers of considerable interest. The first was on the use of collodion for the cure of entropion; the second was on a new instrument for cauterizing the urethra; and the third was on a new apparatus for treating false anchylosis of the knee joint.

Invitations were received during the day for the society to visit the several State institutions located in this place. In the evening, the society assembled with a very respectable number of citizens, to hear the anniversary address from Dr. C. N. Andrews of Rockford. The particular design of the address was to show that the three professions, technically so called, had their origin in the necessities of human society; that they constituted the basis of all proper civilization, and that their relations to each other and to mankind were mutual and co-operative, not antagonistic. It was well written and was listened to with much attention. The society assembled on the morning of the third day, and, after completing the business of the annual session, adjourned to comfortable carriages, and proceeded to visit the institutions for the deaf and dumb, the blind, and the insane; want of time alone preventing them from visiting also the literary college established here. At the two first named institutions we found everything in excellent order, and we should not hesitate to rank them among the best institutions of the kind in our country. The building for the Insane Hospital is a pleasantly located and imposing structure; only a small part of the interior is finished and occupied at present, though the work of completion is rapidly progressing. Dr. Higgins, the superintendent, received the members of the society very cordially, and aside from a short preliminary lecture on good manners towards the insane, rendered their visit both pleasant and profitable. The institution is certainly one of the noblest charities of the State. Individually, we were highly pleased with our visit to Jacksonville—all the doings in the State Society were most pleasant and harmonious; the brethren of the profession located there were most hospitable and kind; and the place itself, with the surrounding country, most beautiful. But enough.

Jacksonville, June 3, 1852, N. S. DAVIS.

